# Cystic fibrosis transmembrane conductance regulator function in patients with chronic pancreatitis

**DOI:** 10.1097/MD.0000000000028904

**Published:** 2022-02-25

**Authors:** Tobias Schlosser, Daniel Fischer, Sandra Büttner, Valentin Blank, Albrecht Hoffmeister

**Affiliations:** a Division of Gastroenterology, Department of Medicine II, Leipzig University Medical Center, Leipzig, Germany; b Outpatient Psychiatry and Psychotherapy, Leipzig, Germany.

**Keywords:** chronic pancreatitis, cystic fibrosis, nasal potential differences, superfusion

## Abstract

**Background:**

Pathogenesis of chronic pancreatitis (CP) is still not entirely understood with many patients probably having more than 1 underlying etiology. Besides toxic-metabolic factors, genetics contribute to disease development. Mutations in cystic fibrosis transmembrane conductance regulator (*CFTR*) are shown to increase risk for CP. Activity of CFTR can easily be accessed in vivo by measurement of nasal potential difference (PD).

**Methods:**

We compared in this monocentric study 17 CP patients from the outpatient unit of our university hospital with 30 healthy controls regarding nasal PDs by using a superfusion protocol. Additionally, demographic and lifestyle data of all persons were recorded.

**Results:**

Seventeen patients (12% female, median age 48 years) with CP and 30 healthy volunteers (47% female, 25 years) were included in the study. Patients with CP had a significant higher proportion of CFTR dysfunction (*P* = .04). Furthermore, demographics differed between the 2 groups with CP patients being older (*P* < .001). There were differences in daily alcohol consumption (*P* = .001) and smoking habits (smokers vs nonsmokers: *P* = .01, pack years: *P* = .002).

**Conclusions:**

PD measurement is an easily accessible way to show CFTR dysfunction as an etiological factor of CP. Cigarette smoking might impair CFTR function and therefore be 1 preventable cause of CP evolution.

## 1 Introduction

Cystic fibrosis (CF) is the most common severe autosomal recessive disease among Caucasian population with an incidence of around 1 to 3000 live births.^[1]^ Underlying genetic base are variable mutations within the gene encoding for CF transmembrane conductance regulator (*CFTR*). These mutations lead to dysfunctional chloride transport, which causes viscous secretions in lungs, pancreas, liver, intestine, and reproductive tract. Most patients develop multisystem disease involving several or all mentioned organs.

CF is associated with different pancreatic diseases, such as exocrine and/or endocrine insufficiency.^[2–4]^ However, acute pancreatitis is a rather infrequent complication of CF (1.24% of all CF patients) and usually occurs in patients with pancreatic sufficiency (PS). Nevertheless, in CF patients with PS an episode of acute pancreatitis is quite frequent (10%–17% of CF patients with PS).^[5,6]^

Chronic pancreatitis (CP) is a syndrome involving pancreatic inflammation, fibrosis, and loss of function. These conditions lead to a variety of clinical manifestations such as chronic abdominal pain, steatorrhea and maldigestion promoted by exocrine insufficiency, and pancreatogenic diabetes promoted by endocrine insufficiency.^[7–9]^ Annual incidence of CP varies heavily between 1.6 and 23/100.000 persons.^[10]^ Etiologic classification subsets toxic-metabolic, idiopathic, genetic, autoimmune, recurrent acute pancreatitis, and obstructive causes (“TIGAR-O”).^[11]^ Within the toxic etiologies, alcohol and nicotine abuse represent by far the most dominant risk factors for CP in western countries.^[10]^

Diagnosis of CP is based upon clinical conditions, function testing, and typical findings on imaging studies.^[9,12]^ Irreversible tissue damage can be assessed as morphological changes by distinct and complementary modalities. Percutaneous and endoscopic ultrasound reveal characteristic CP patterns as well as cross-sectional imaging (CT [computed tomography], MRI [magnetic resonance imaging] / MRCP [magnetic resonance cholangiopancreatography]). Pancreatic calcification as 1 pathognomonic feature may even be visible on plain abdominal radiograph. The Cambridge classification helped to score and unify nomenclature of different imaging techniques and is also part of the M-ANNHEIM scoring system for CP severity.^[13,14]^ ERCP has lost its diagnostic role to the mentioned noninvasive methods and is reserved for therapeutical use. Histopathological proof as gold standard in CP diagnosis is rarely needed nowadays (eg, to establish diagnosis of autoimmune pancreatitis according to the HISORt criteria).^[15,9,12]^

Therapy of CP consists of pain management, preservation of pancreatic sufficiency or correction of insufficiencies, and management of complications with malignant transformation being one of the severe sequela.^[12,9]^

A presumed connection between CF and CP was first described in 1969: 2 siblings with CF were born to a family with 3 paternal relatives suffering from CP.^[16]^ In the 1980s *CFTR*-mutation was identified as the genetic base of CF.^[17]^

Due to histopathological (“Cystic fibrosis is basically a diffuse form of chronic pancreatitis.”^[18]^) and clinical resemblance between CF and CP, Cohn et al^[19]^ and Sharer et al^[20]^ and hypothesized and revealed connection between CFTR dysfunction and CP. Indeed, mutations within the *CFTR*-gene can cause CP with or without manifestations of CF depending on mutation type and zygosity. Thousands of genetic *CFTR*-variations are known and influence the clinical course of related diseases.^[21,22]^ Acute recurrent or CP episodes may precede CF diagnosis.^[23]^ Evolution to CP in homozygous CF patients rarely happens. In a large multinational study only 10 out of 10.071 CF patients had CP.^[5]^ Heterozygous carriers of *CFTR*-mutations (eg, parents of CF patients) have a 3- to 4-fold risk of developing CP.^[24]^ Genetic analysis of 134 CP patients (71 alcohol related, 60 idiopathic) revealed at least 1 abnormal *CFTR*-allele in 18 (13,4%) individuals. These subgroup showed significant low nasal potential-difference values without reaching diagnostic threshold for CF.^[20]^ A case-control study presented around twice as much *CFTR*-mutations (18.6% vs 9.2%, *P* < .05) in 67 patients suffering from idiopathic CP compared to 60 healthy individuals.^[24]^ A similar study found an over 6-fold ratio of dysfunctional *CFTR*-genes in patients with undetermined acute, recurrent, or CP (19 of 96 cases, 19%, as compared to 7 of 198 controls, 3.5%; *P* < .00001).^[25]^

Given *CFTR*-mutations as a risk factor for CP, aim of this study was to analyze whether CP is associated with CFTR-dysfunction, measured by nasal potential difference (PD). This method is established as the most sensitive test for CFTR dysfunction in patients suspicious for CF. Furthermore, the influence of smoking regarding CFTR-function in CP was evaluated.

## 2 Methods

A total of 17 patients with CP had been recruited at the Leipzig University Hospital and examined by nasal PD measurement. Results were compared to the measurements of 30 healthy controls, leading into an unmatched case control study.

Diagnosis of CP were established by an experienced gastroenterologist. Participants had to provide insight of their medical records regarding the exclusion of pancreatic diseases. Missing values were completed by a self-reported questionnaire. Smokers identified themselves without numeric threshold. Alcohol intake was reported on an estimated daily base. Questions referred to the current nicotine and alcohol consumption at the time of the study. Referring to cigarette consumption, pack years (PY) were defined as packs of 20 cigarettes per day multiplied by total years of smoking.

The study was performed in accordance with the guidelines for good clinical practice (E6/R1) and the ethical guidelines of the Helsinki Declaration and was approved by the local ethics committee (University of Leipzig). Informed written consent was obtained from all participants. Neither financial nor nonfinancial benefits were given in return for participation.

PD measurements were conducted according to protocol by Schüler et al^[26]^: The test person laid his head upon an ophthalmic chin rest while leaning forward to allow the perfusing liquids to exit the nostril. The first in vivo electrode (Ag/AgCl electode, Fa. World Precision Instruments [WPI], Friedberg, Germany) was placed by the operator under visual guidance into 1 nostril under the inferior turbinate contacting nasal mucosa. This exploring electrode was then secured by tape (Leukosilk S, Fa. BSN Medical, Germany). The second electrode connected a needle (Butterfly BD Valu-Set, Fa. Becton Dickinson Industries, NJ) that had been placed subcutaneously in the person's forearm and served as reference. Basic PD of nasal mucosa was initially measured with a NaCl-perfusion (0.9% sodium chloride, Fa. Baxter, IL). Subsequently followed by measurement under nasal perfusion with amiloride in order to inhibit local sodium channels. Finally, a nonchloride perfusion and perfusion with salbutamol stimulated CFTR function (“superfusion”). Solutions were compounded by the authors especially for this study (Table A1, Supplemental Digital Content). Agar (3%) in Ringer's solution served as bridging device for the measuring electrodes. PD was graphically presented via voltmeter (Isolated Biological Amplifier, Fa. WPI) and recorded by Data-Trax Data Aquisition Software (Fa. WPI). CFTR dysfunction was defined by missing response to superfusion as indicated by more positive values after salbutamol-perfusion compared to initial PD (Figure A1, Supplemental Digital Content, supplemental content, presenting exemplary curves). Intact nasal mucosa (without, eg, acute rhinitis or recent surgical intervention) was precondition for every measurement.

Statistics and graphics were computed with open access software “R”, version 4.0.4 (The R Foundation for Statistical Computing Vienna, Austria). Chi-squared (Chi^2^) testing for binary variables and 2-sided *t* test for continuous variables checked for inequality between compared groups. Welch modification of *t* test was used for inhomogeneity of variance, which had been previously tested via Levene test.^[27]^*P*-values below 5% served as criterion toward significance.

## 3 Results

### 3.1 Patients characteristics

A total of 17 patients (15 men, 2 women) with CP had been prospectively recruited at the Leipzig University Medical Center. Main characteristics are shown in Table 1. The patients had a median age of 48 years (range 31–73 years) at study entry. Fourteen patients (82%) reported a mean alcohol uptake of 60 g per day; 3 patients (18%) claimed no regular alcohol consumption. Eleven CP patients (65%) were self-reported smokers, whereas 6 (35%) didn’t smoke.

**Table 1:** Patient characteristics.

	Chronic pancreatitis		Healthy control			
Characteristic	No.	%	No.	%	Chi^2^	*t* test
Total	17	100%	30	100%		
Etiology						
Alcohol	7	41%				
Hereditary	1	6%				
Idiopathic	4	24%				
Unknown	5	29%				
Age at diagnosis [yr]						
Median (range)	41 (3–64)					
Age at test [years]						<0.001^∗∗∗^
Median (range)	48 (31–72)		25 (22–73)			
Gender						
Male	15	88%	16	53%	.04^∗^	
Female	2	12%	14	47%		
Alcohol [g/d]						0.001^∗∗∗^^,^^†^
Median (range)	60 (0–200)		9 (0–20)			
No alcohol	3	18%	7	23%	.93	
Smoking [pack yr]						0.002^∗∗^^,^^†^
Median	11		0			
Range	0–30		0–10,5			
Smoker	11	65%	7	23%	0.01^∗^	
Nonsmoker	6	35%	23	77%		
CFTR						
Dysfunction	6	35%	2	7%	0.04^∗^	
Normal	11	65%	28	93%		

CFTR = cystic fibrosis transmembrane conductance regulator, g/d = gram per day.

† Welch modification.

∗ *P* < .05.

∗∗ *P* < .01.

∗∗∗ *P* < .001.

Thirty healthy volunteers (16 men, 14 women) served as control group and presented with a higher female proportion compared to CP patients (*P* = .04). The median age was 25 years (range 22–73 years) and the subjects were significantly younger than the CP patients (*P* = .001). Daily alcohol intake ranged from 0 to 20 g with a mean intake of 9 g. Less alcohol consumption was noted (*P* < .001) within the control group. Seven subjects (23%) were smokers and 23 persons (77%) were nonsmokers, showing a higher number of smokers in the CP group (*P* = .01).

### 3.2 Potential differences (PD) and CFTR function

**Figure 1:**
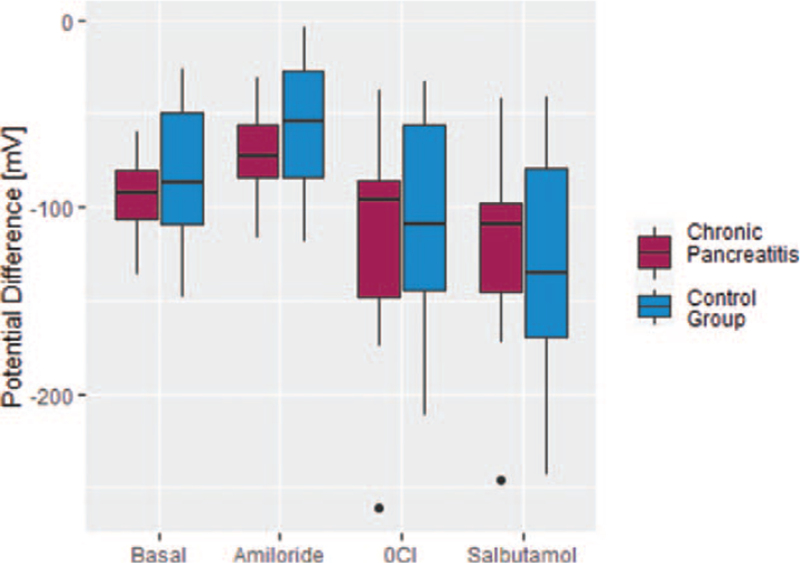
Grouped boxplots of potential differences under perfusion with NaCl (basal), amiloride, chloride-free solution (0Cl) and salbutamol. Median values are denoted by horizontal bar. Dots represent outliners.

Mean values of PD are provided in Table 2 and shown as boxplots in Figure 1. CP patients presented with a baseline PD of −93.2 mV (± 22.3 mV) compared to −83.7 mV (± 35.1 mV) in healthy volunteers. Amiloride perfusion resulted in raising PDs toward −70.1 mV (± 25 mV) in patients with CP and −57.4 mV (± 33.1 mV) in healthy volunteers, respectively. Excluding chloride of the perfusions lowered PDs of CP patients to −120 mV (± 51.9 mV) and of control persons to −107 mV (± 53.3 mV). Adding salbutamol lead to a further fall of PD with mean values of −123 mV (± 47.2 mV) in CP patients and −128 mV (± 56.3 mV) in healthy test persons. Basal potentials as well as all perfusion solutions did not differ significantly between CP and control group (exact *P*-values ranged from .18–.76, Table 2).

**Table 2:** Nasal potential differences.

	Chronic pancreatitis	Healthy control	
PD [mV]	Mean (± SD)	Mean (± SD)	*t* test
Baseline	−93.2 (± 22.3)	−83.7 (± 35.1)	0.26^∗^
Amiloride	−70.1 (± 25.0)	−57.4 (± 33.1)	0.18
Chloride-free	−120 (± 51.9)	−107 (± 53.3)	0.42
Salbutamol	−123 (± 47.2)	−128 (± 56.3)	0.75

mV = miliVolt, PD = nasal potential differences, SD = standard deviation.

∗ Welch modification.

**Figure 2:**
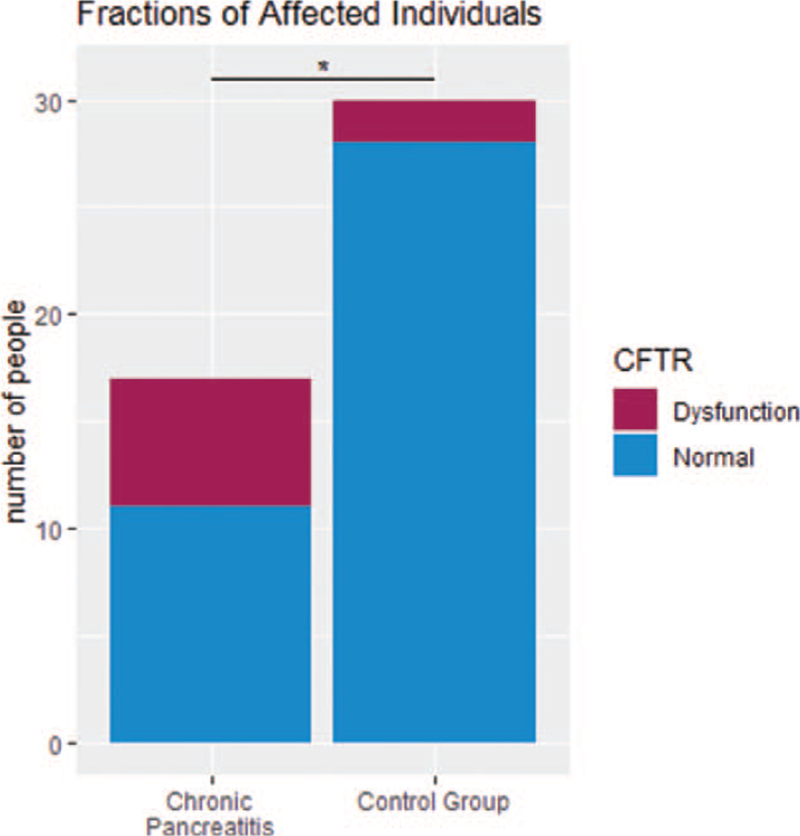
Stacked bar charts of CFTR function within patients with chronic pancreatitis compared to healthy control group. CFTR = cystic fibrosis transmembrane conductance regulator.

Only 2 out of 30 healthy controls (7%) showed decreased response to stimulation, whereas 6 out of 17 (35%) patients with CP presented a reduced salbutamol-response as indicator for CFTR dysfunction (*P* = .04, Table 1, Fig. 2, see Figure A2, Supplemental Digital Content, presenting patient characteristics regarding CFTR function, see Table A3, Supplemental Digital Content, presenting PDs regarding CFTR function). Logistic regression shows an odds ratio of 2.03 (standard error 0.89, *P* = .02), that a person with CFTR dysfunstion had been diagnosed with CP.

Within the 17 CP patients were 5 smokers, who presented CFTR dysfunction, and 6 smokers with normal CFTR function. The remaining 6 nonsmokers could be separated into 1 CP patient with CFTR dysfunction and 5 with physiological CFTR function. These groups did not show statistical significance regarding chi-squared test (Table 3, *P* = .51).

**Table 3:** Smoking and CFTR function in CP patients.

	CFTR dysfunction	CFTR normal	Chi^2^	*t* test
Median of daily cigarette consumption	16	8		0.180
Median of cigarette pack years	22,5	2,5		0.042^∗^
Smoker	5	6	0.51	
Nonsmoker	1	5		

CFTR = cystic fibrosis transmembrane conductance regulator, CP = chronic pancreatitis.

∗ *P* < .05.

**Figure 3:**
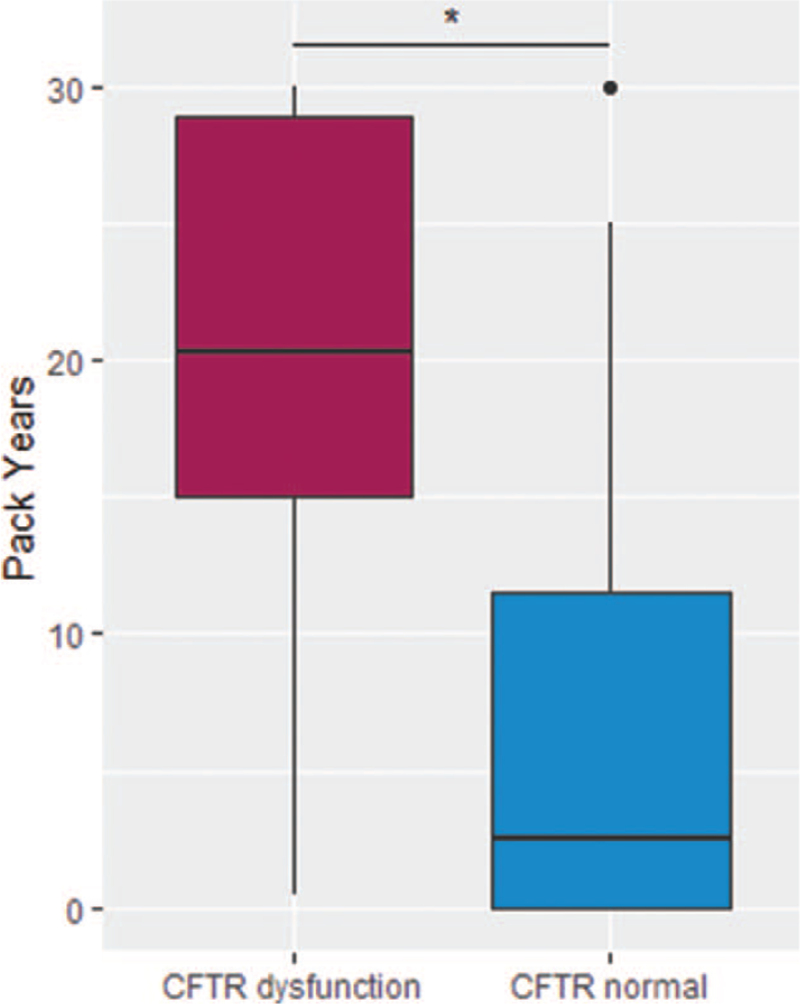
Boxplots of cigarette consumption as pack years and CFTR function within patients with chronic pancreatitis. Median values are denoted by horizontal bar. Dots represent outliners. CFTR = cystic fibrosis transmembrane conductance regulator.

Comparing the amount of smoking on a daily base showed that CP patients with CFTR dysfunction had a median cigarette consumption of 16 per day, whereas these with normal CFTR function smoked 8 cigarettes per day (*P* = .18). Median amount of PY was significantly associated with CFTR dysfunction (22.5 vs 2.5 PY, *P* = .04, Fig. 3, Table 3) within CP patients. This observation disappears when looking at the entire cohort (study group plus healthy volunteers combined, *P* = .25, Table 4).

**Table 4:** Smoking and CFTR function in CP and non-CP patients.

	CFTR dysfunction	CFTR normal	Chi^2^
Smoker	5	13	0.25
Nonsmoker	3	26	

CFTR = cystic fibrosis transmembrane conductance regulator, CP = chronic pancreatitis.

## 4 Discussion

Exact mechanisms of CP development are not entirely understood. One theory is that proteins precipitate and obstruct pancreatic ducts, thus leading to atrophy.^[4]^ Based on that hypothesis, viscous ductal fluid due to decreased CFTR function might be 1 key point in pathogenesis of CP.^[28]^

Malfunctioning CFTR had been observed in distinct etiologies such as idiopathic, alcoholic and autoimmune CP.^[29]^ Cigarette smoke does suppress CFTR function in vitro as well as in vivo, observed by nasal PD measurement of smokers without genetic alterations.^[30]^ Our data support that idea as we show significant decrease of CFTR activity in nasal PD measurements within the CP cohort (Table 1) but not within the entire cohort (Table 4). This could represent 1 hint, that CP patients might present a special pathogenic susceptibility towards tobacco use. Furthermore, there seems to be a dose depending effect as the consumed PY show effect on CFTR function, but not the daily number of cigarettes (Table 3). These 2 observations combined might support our idea that cigarette smoke contributes to pathogenesis of CP via interfering CFTR activity.

Exact mechanism of decreased CFTR activity in smokers is yet not established. Internalization of the channel due to cigarette consumption might be 1 explanation that was derived from in vitro experiments.^[31,32]^ Direct CFTR inhibition by reactive smoke intermediates might as well contribute.^[33]^ Furthermore, smoke leads to degradation of CFTR and might also contribute to associated diseases.^[34]^ Besides smoking cessation, there might also be a pharmacological approach to re-establish CFTR function with therapeutic intention.^[35–37]^

Therefore, we hypothesize that cigarette consumption as risk factor for CP development might be mediated by smoking-induced CFTR dysfunction.

Our study results have some limitations that must be taken into consideration: Change of smoking habits or in alcohol consumption over time were not recorded. Especially when diagnosed with CP you might expect cessation or reduction of these well-known risk factors according to medical advice. Besides, the self-report of these habits might tend to be biased by desirability in order to dissimulate unhealthy behavior and simulate medical compliance.^[38]^ Moreover, self-assessment of data is lacking objectiveness and tend to include systemic bias.^[39]^ Another limitation of the study is the heterogeneity of the investigated groups. This is mainly a result of a selection bias within the unmatched case control design of our study that is lacking a stratified randomly sampled control group. Nevertheless, the differences between the groups seem quite reasonable: Although there is a well-known “gender gap” in CF as role model of CFTR associated disease, genetics lead to an equally balanced distribution of CFTR alleles in both genders. Disparities of CF diagnosis and course of disease are related to many factors and are not fully understood.^[40]^ Higher male proportion of our CP study group is consistent with higher incidence of CP in men (55%–91%).^[41–43]^ It remains unclear whether there are genetic risk factors for men regarding CP evolution.^[41]^ The more severe tobacco and alcohol consumption of men might have obvious influence and is mirrored in higher male ratio when epidemiology is linked to toxic lifestyle factors.^[41,44,45]^ Within our group of CP patients were more smokers and persons with higher consumption of alcohol (Table 1). This finding supports, that these established risk factors agents^[46]^ contributed to disease development in our CP cohort as well.

CP patients were of older age at time of PD measurement (Table 1). As its function is mostly determined by germ line genetics, an effect of ageing on CFTR activity is unlikely. Nevertheless, a dysfunction of CFTR due to prevailing life span might be interpreted as cumulative environmental influences over time. Moreover, we supported this theory already by the correlating time effect of cigarette PY (Table 3).

Altogether CFTR dysfunction could be associated with CP. Nasal PD measurement is an easy way to identify altered CFTR function and may help determine the etiology of CP without need for genetic sequencing.

## Acknowledgments

We acknowledge support from Leipzig University for Open Access Publishing.

## Author contributions

**Conceptualization:** Tobias Schlosser, Daniel Fischer, Sandra Büttner, Albrecht Hoffmeister.

**Data curation:** Tobias Schlosser, Daniel Fischer, Sandra Büttner.

**Formal analysis:** Tobias Schlosser.

**Investigation:** Tobias Schlosser, Daniel Fischer, Sandra Büttner, Valentin Blank, Albrecht Hoffmeister.

**Methodology:** Tobias Schlosser, Valentin Blank, Albrecht Hoffmeister.

**Project administration:** Albrecht Hoffmeister.

**Resources:** Tobias Schlosser, Albrecht Hoffmeister.

**Software:** Tobias Schlosser.

**Supervision:** Albrecht Hoffmeister.

**Visualization:** Tobias Schlosser.

**Writing – original draft:** Tobias Schlosser.

**Writing – review & editing:** Valentin Blank, Albrecht Hoffmeister.

## Supplementary Material

Supplemental Digital Content
