# Supplementary material for: Cystic fibrosis transmembrane conductance regulator function in patients with chronic pancreatitis
Source: Medicine (Baltimore). 2022 Feb 25;101(8):e28904. doi: 10.1097/MD.0000000000028904 (PMC8878632; doi:10.1097/MD.0000000000028904)
Supplement: Supplemental Digital Content [file medi-101-e28904-s001.docx]

Appendix


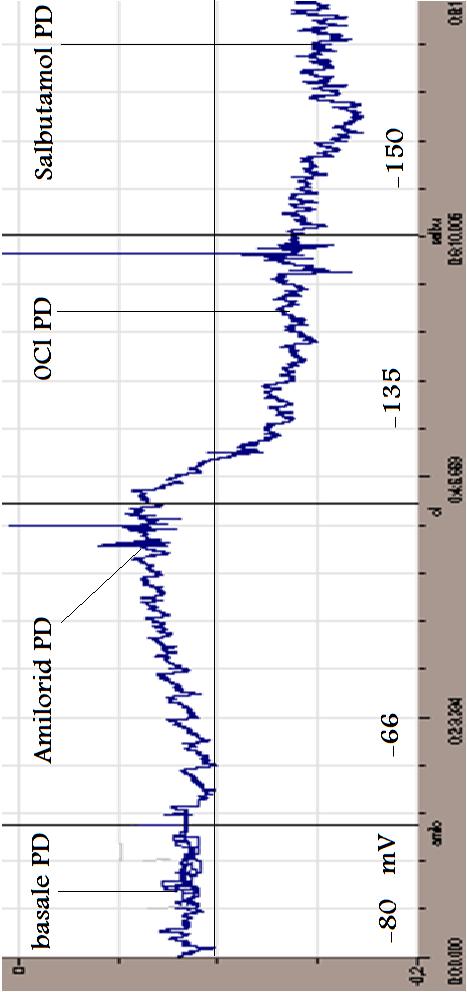


**A**


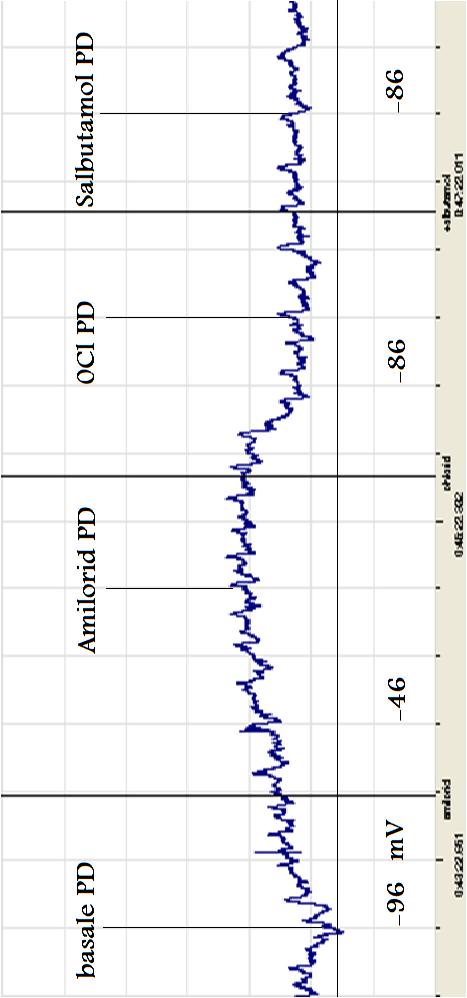


**B**

**Figure A1**. Exemplary curves. A. Physiological CFTR function: Application of salbutamol shows decrease of potential difference (PD) below initial (=basal) value. B. CFTR dysfunction: Superfusion with salbutamol doesn´t result in adequate response. Red lines denote basal PD.

*0Cl: chloride-free solution, CFTR: cystic fibrosis transmembrane conductance regulator*

| ***Table A1.*** *Detailed compounds of 1000 ml amiloride solutions* | | | | | |
| --- | --- | --- | --- | --- | --- |
| ***+ amiloride***  ***+ chloride***  ***- salbutamol*** | | ***+ amiloride***  ***- chloride***  ***- salbutamol*** | | ***+ amiloride***  ***- chloride***  ***+ salbutamol*** | |
| K_2_HPO_4_ | 418 mg | K_2_HPO_4_ | 418 mg | K_2_HPO_4_ | 418 mg |
| KH_2_PO_4_ | 55 mg | KH_2_PO_4_ | 55 mg | KH_2_PO_4_ | 55 mg |
| NaCl | 6 721 mg | NaCl |  | NaCl |  |
| Na gluconate |  | Na gluconate | 25 081 mg | Na gluconate | 25 081 mg |
| NaCO_3_ | 2 100 mg | NaCO_3_ | 2100 mg | NaCO_3_ | 2 100 mg |
| MgCl_2_ | 244 mg | MgCl_2_ | - | MgCl_2_ | - |
| MgSO_4_ | - | MgSO_4_ | 296 mg | MgSO_4_ | 296 mg |
| CaC_l2_ | 133 mg | CaC_l2_ | - | CaC_l2_ | - |
| Ca gluconate | - | Ca gluconate | 533 mg | Ca gluconate | 533 mg |
| Amiloride | 30 mg | Amiloride | 30 mg | Amiloride | 30 mg |
| Salbutamol | - | Salbutamol | - | Salbutamol | 2.9 mg |
| *ml: millilitre, mg: milligram, mol: imole, + : included, - : excluded* | | | | | |

| ***Table A2.*** *Patient characteristics* | | | | | | | | |  |  |
| --- | --- | --- | --- | --- | --- | --- | --- | --- | --- | --- |
|  | | | ***CFTR dysfunction*** | | | ***CFTR normal*** | | | ***Chi²*** | ***t-test*** |
| Characteristic | | | No. |  | % | No. |  | % |  |  |
| Total | | | 8 |  | 100% | 39 |  | 100% |  |  |
| Age [Years] | | |  |  |  |  |  |  |  | 0.04* |
|  | | Median |  | 47 |  |  | 26 |  |  |  |
|  | | Range |  | 24-72 |  |  | 22-73 |  |  |  |
| Gender | | |  |  |  |  |  |  | .85 |  |
|  | | Male | 6 |  | 75% | 25 |  | 64% |  |  |
|  | | Female | 2 |  | 25% | 14 |  | 36% |  |  |
| Alcohol [g/d] | | |  |  |  |  |  |  |  | 0.20*⟡* |
|  | | Median |  | 60 |  |  | 10 |  |  |  |
|  | | Mean |  | 68 |  |  | 22 |  |  |  |
|  | | Range |  | 0-200 |  |  | 0-200 |  |  |  |
|  | | No Alcohol | 1 |  | 13% | 9 |  | 23% | 0.85 |  |
| Smoking | | |  |  |  |  |  |  |  |  |
|  | | Yes | 5 |  | 63% | 13 |  | 33% | 0.25 |  |
|  | No | | 3 |  | 38% | 26 |  | 66% |  |  |
| *⟡: Welch modification, * : p < 0.05, **: p < 0.01, ***: p < 0.001, CFTR: cystic fibrosis transmembrane conductance regulator* | | | | | | | | | | |

| ***Table A3.*** *Nasal potential differences* | | | | |
| --- | --- | --- | --- | --- |
|  | | ***CFTR dysfunction*** | ***CFTR normal*** |  |
| PD [mV] | | Mean (± SD) | Mean (± SD) | t-test |
| Baseline | | -102 (± 23.4) | -84.1 (± 31.9) | 0.14 |
| Amiloride | | -68.0 (± 28.5) | -60.8 (± 31.4) | 0.55 |
|  | Chloride-free | -76.8 (± 27.0) | -118 (± 54.0) | 0.004**⟡ |
|  | Salbutamol | -82.5 (± 29.0) | -135 (± 52.6) | 0.009** |
| *PD: Nasal potential differences, mV: miliVolt, SD: standard deviation, ⟡: Welch modification, * : p < 0.05, **: p < 0.01, ***: p < 0.001, CFTR: cystic fibrosis transmembrane conductance regulator* | | | | |
